# Development of an Implementation Blueprint to Scale-Up Contraception Care for Adolescents with Psychiatric Conditions in a Pediatric Hospital

**DOI:** 10.1007/s43477-023-00082-7

**Published:** 2023-05-12

**Authors:** Kathryn A. Hyzak, Alicia C. Bunger, Samantha A. Herrmann, Anna Kerlek, Stephanie Lauden, Sam Dudley, Abigail Underwood, Elise D. Berlan

**Affiliations:** 1 College of Social Work, The Ohio State University, 1947 College Road, Columbus, OH 43215 USA; 2 Department of Psychiatry and Behavioral Health, Nationwide Children’s Hospital, Columbus, OH USA; 3 The Ohio State University College of Medicine, Columbus, OH USA; 4 Division of Hospital Medicine, Nationwide Children’s Hospital, Columbus, OH USA; 5 Department of Pediatrics, The Ohio State University College of Medicine, Columbus, OH USA; 6 Division of Adolescent Medicine, Nationwide Children’s Hospital, Columbus, OH USA; 7 Division of Pediatric Hospital Medicine, Children’s Hospital Colorado, Aurora, CO United States

**Keywords:** Implementation blueprint, Contraception, Hospitals, Pediatric, CFIR

## Abstract

Implementation blueprints are comprehensive plans that describe implementation strategies, goals, timelines, and key personnel necessary for launching new interventions. Although blueprints are a foundational step in driving intervention rollout, little is known about how blueprints are developed, refined, and used in practice. The objective of this study was to describe a systematic, collaborative approach to developing, refining, and utilizing a formal implementation blueprint for scaling up the Contraception Care at Behavioral Health Pavilion (CC@BHP) intervention for adolescents hospitalized in psychiatric units within a pediatric hospital in the United States. In Stage 1 (Planning/Preparation), we assembled a Research Advisory Board (RAB) of 41 multidisciplinary members and conducted a formative evaluation to identify potential barriers to CC@BHP implementation. Barriers were mapped to implementation strategies using the Consolidated Framework for Implementation Research (CFIR) and Expert Recommendations for Implementing Change (ERIC) tool and used to create an initial blueprint. In Stage 2 (Development/Implementation), RAB members used activity logs to track implementation activities over the 18-month study period, which were then mapped to formal implementation strategies used to further develop the blueprint. About 30% of strategies were situated in the ‘Train and Educate Stakeholders’ ERIC category, 20% in ‘Use Evaluative and Iterative Strategies,’ and 16% in ‘Develop Stakeholder Interrelationships’ category. In Stage 3 (Synthesis/Refinement), the final blueprint was refined, consisting of 16 goals linked to 10 strategies for pre-implementation and 6 strategies for implementation. Feedback on the blueprint emphasized the role of the project champion in translating the blueprint into smaller, actionable steps for implementers.

**Supplementary Information:**

The online version contains supplementary material available at 10.1007/s43477-023-00082-7.

## Introduction

Creating an implementation plan, or blueprint, is a foundational step to prepare for launching a new program, model, or intervention (Aarons et al., 2011). A blueprint is a formal implementation plan that describes the specific goals, strategies, units, and/or personnel involved in the implementation change process, and timeframes for completion of goals to aide in the integration of an evidence-based practice or program into routine care (Powell et al., 2012, 2015). Implementation blueprints are guides for leaders and practitioners to enhance readiness and support intervention scale-up across different sites. In the context of implementation research, blueprints can also be used to track, specify, and report implementation strategies, which is important for promoting transparency and understanding impact on implementation outcomes (Powell et al., 2019).

Implementation blueprint development processes can vary—some might be designed as a structured and robust research-driven process (e.g., Lewis et al., 2018), while other blueprint development processes draw more on community engagement (e.g., Valentine et al, 2022). Despite the potential usefulness of implementation blueprints, little attention has been paid to how implementation blueprints are developed or used in practice or research. To address this gap, this study describes the processes we used to develop, refine, and apply a formal implementation blueprint to launch a new contraception care intervention in a pediatric hospital setting. Our blueprint can be adapted by other hospital settings to aide in the implementation of the intervention in those settings, or these methods can be replicated to develop an implementation blueprint for other intervention implementation purposes.

## Background

### Developing, Refining, and Using Implementation Blueprints

As defined in the original implementation strategy taxonomy, an implementation blueprint is a formal plan that “integrates multiple strategies from multiple levels or domains… using multiple theories or the use of an explicit theoretical framework” (Powell et al., 2012, p. 14). Blueprints specify measurable objectives, strategies, and timing of change efforts (Powell et al., 2015). Importantly, blueprints offer detail about how implementation strategies will be or were operationalized in a particular setting, including specific action steps, persons responsible, and the level of importance of each goal (Lewis et al., 2018; Valentine et al., 2022). Developing a written implementation plan or blueprint is one of the main expected outcomes during preparation (Aarons et al., 2011) or pre-implementation phases (Saldana, 2014).

Blueprints reflect systematic planning and deliberate efforts to tailor implementation strategies to the local context (Powell et al., 2012, 2015). As described in Lewis et al. (2018) and Valentine et al. (2022), implementation blueprint development is a collaborative process that engages members who will be responsible for implementing an innovation (e.g., supervisors, clinicians), those who will have a role in supporting implementation or removing implementation barriers (e.g., key agency/unit leaders, quality improvement specialists), and ideally, people who will be benefitting from the innovation (i.e., patients and caregivers) (Lewis et al., 2018; Valentine et al., 2022). Multiple member involvement in the blueprint development process has potential to build buy-in for implementation, enhance fit, and improve the likelihood of successful implementation.

Several common elements reflect a systematic planning approach to developing an implementation blueprint, as detailed in Lewis et al. (2018) and Valentine et al. (2022). First, blueprint development begins with an identification or assessment of barriers and facilitators. This assessment might be based on surveys or focus groups conducted as part of pre-implementation work (Lewis et al., 2018) and/or conversations and workgroup meetings (Valentine et al., 2022). Next, this information, along with an implementation framework or theory, is used to guide analysis of these barriers and facilitators, as well as to select implementation strategies that deliberately address the barriers and leverage facilitators. For instance, Lewis et al. (2018) used the Framework for Dissemination to guide their mixed methods data collection and analyses of potential determinants to implementation of a cognitive behavioral therapy intervention in a youth residential treatment center, and then selected strategies from an existing, published compilation of strategies to inform blueprint development. Third, members work together to operationalize implementation strategies in the local context, where implementation strategies are tailored differently depending on the needs of the setting (Lewis et al., 2018; Valentine et al., 2022). For instance, Valentine et al. (2022) described their process for stakeholder engagement in the implementation strategy selection process, where they assembled a community advisory board to aide in the selection and application of implementation strategies to rollout out an intervention for post-traumatic stress disorder treatment in primary care. Operationalization or specification should provide replicable details about who, when, and where implementation strategies will be used (Proctor et al., 2013).

These operational details could be helpful for planning and generating a shared understanding of how an innovation will be implemented. However, as with most plans, few unfold exactly as expected. Especially in implementation, unexpected challenges arise requiring implementers to assess the situation, deploy implementation strategies in real time, and make blueprint adjustments (Bunger et al., 2017). Therefore, implementation blueprints should be refined to include and specify the implementation strategies used.

Implementation strategy-tracking tools have potential to capture information about the implementation strategies used in practice (Boyd et al., 2018; Walsh-Bailey et al., 2021). This could be important for evaluating the use of pre-planned implementation strategies (i.e., did we use the strategies we thought we needed?), identifying unplanned implementation strategies used to address emergent challenges, and capture greater detail that would allow specification (Garcia et al., 2022). Accurate and detailed specification of the implementation strategies in a blueprint have potential to support replication and scale up in other units and sites (Centers for Disease Control & Prevention, 2016). However, further investigation is needed to understand how implementation blueprints are developed, whether and how they are used, and by whom. Our aim was to investigate this gap, and therefore, we sought to develop, refine, and pilot an implementation blueprint. This work was conducted in the context of a contraception care consultation intervention in a complex, pediatric hospital setting.

### The Context: Contraceptive Care Clinical Consultation

At the intervention site, adolescents hospitalized for mental health conditions are cared for across several clinical units and their general medical care is provided by the pediatric hospitalist service (HP). The Contraception Care at Behavioral Health Pavilion (CC@BHP) intervention trained HP advance practice providers (APPs) to deliver contraception care to enhance adolescents’ access to contraception. The CC@BHP intervention included the following components: (1) assessing interest in contraception care, (2) placing a consultation for contraception care, (3) delivering contraception counseling, and (4) providing contraceptives (including etonogestrel implants) to interested adolescents hospitalized with psychiatric disorders.

Adolescents experience many barriers to accessing contraception including cost, privacy, and logistical constraints (Brittain et al., 2018). Mental health disorders have been associated with inconsistent contraceptive use, use of less effective methods, contraceptive non-use, and discontinuation of contraception (Hall et al., 2013, 2015; Stidham Hall et al., 2013). Importantly, a link exists between symptoms of mental health disorders, particularly depressive symptoms, and unintended pregnancy (James-Hawkins et al., 2014). Avoiding an unintended pregnancy is particularly important for female adolescents with underlying mental health conditions given associations with exacerbation of underlying mental health disorders and adverse pregnancy-related outcomes (Bahk et al., 2015; Boden et al., 2015). Current birth control methods allow patients with psychiatric conditions to delay pregnancy until their mental health care is optimized allowing for safest reproductive outcomes (McCloskey et al., 2021). Furthermore, ensuring adolescents have access to contraception is consistent with policies of the American Academy of Child and Adolescent Psychiatry (American Academy of Child & Adolescent Psychiatry, 2022).

Inpatient psychiatric hospitalization presents a unique and underutilized opportunity to provide reproductive healthcare to adolescents because co-location of services may be an effective method for reaching these adolescents (Judge-Golden et al., 2018). However, assessing and caring for the sexual health needs of hospitalized adolescents are not commonly performed (Clary et al., 2020; Masonbrink et al., 2018). Studies of hospitalized adolescents have found that they are often interested in learning about sexual and reproductive health and in receiving sexual and reproductive interventions in the hospital setting regardless of age, gender, and prior sexual experiences (Guss et al., 2015; Riese et al., 2019). Therefore, this study sought to provide access to contraception care for adolescents hospitalized with psychiatric conditions who may be at greater risk for poor outcomes should they become pregnant.

The objective of this study was to describe a systematic collaborative approach to developing, refining, and utilizing a formal implementation blueprint for scaling-up the CC@BHP intervention for adolescents hospitalized in psychiatric units within a pediatric hospital located in the Midwestern United States.

## Methods

### Design and Setting

We used the Strengthening the Reporting of Observational Studies in Epidemiology (STROBE) checklist for reporting on all components of this study (Supplemental File 1). This was a prospective cohort study identifying and specifying implementation strategies which we used to inform the development of a tailored implementation blueprint to scale up the CC@BHP intervention across psychiatric units located within the pediatric hospital. Four units were selected as part of a staged intervention rollout process. Units were the (1) Inpatient Psychiatric Unit (IPU) for adolescents hospitalized with psychiatric conditions; (2) Hospital Pediatrics Observation Unit (HPO) service for adolescents admitted for psychiatric conditions under observation and/or who are waitlisted for admission; (3) Youth Crisis Stabilization Unit (YCSU) which provides intensive, individualized psychotherapy and medical care over a three to five day admission; and the (4) Psychiatric Crisis Department Extended Observation Care Unit (EOS). IPU was selected to serve as the pilot unit for intervention implementation and initial blueprint development which subsequently operated as the foundation for the further development and refinement of the final implementation blueprint. We chose to begin rollout on the IPU for two reasons. First, the adolescents served on this unit have a longer length of stay which allows for more time to complete contraception consultations. Second, we had the strongest support from clinicians and leadership who wished to trial the intervention on this unit first. Rollout of the intervention was then followed by HPO, YCSU, and then EOS. Hence, the blueprint was developed and refined over the course of intervention rollout across these four units. This study was approved by the Institutional Review Board at Nationwide Children’s Hospital.

### Procedures

The development, utilization, and refinement of our implementation blueprint took place in three main stages: (1) Planning and Preparation, (2) Development and Implementation, and (3) Synthesis and Refinement. We describe each of these phases in detail below.

### Author’s Positionality Statement

First, we acknowledge and describe the authors’ positionalities regarding the research conducted and framing of our results. Two authors are PhD-level trained Implementation Scientists (KH, AB) and one is a PhD student with a focus on implementation science (AU). These authors are also trained in social work research methods, social work clinical practice, and policy. One is a senior-level academic at the partnering university (AB). Six authors have research and/or clinical expertise in contraception care, contraception care access, and reproductive health and healthcare justice (SH, AK, SL, SD, AU, EB). Four authors are physicians (AK, SL, SD, EB), and one is an advanced practice provider (SH) with expertise in adolescent healthcare in psychiatry or hospital pediatrics. The study Principal Investigator (EB) is a senior-level academic, adolescent medicine physician, and the Director of the adolescent contraception care clinic at the children’s hospital. All authors were involved in the development and/or utilization of the implementation blueprint described in this study, and all are members of the Research Advisory Board for the project.

#### Stage 1: Planning and Preparation

We assembled a Research Advisory Board (RAB) to engage and collaborate with a multidisciplinary team of individuals responsible for guiding intervention implementation. Potential RAB members were recruited by email between May 2021 and September 2022. Members volunteered who had expertise in delivering adolescent psychiatric, medical, pharmacy, and contraception care; quality improvement and organizational change; and individuals who have experienced the hospital system as consumers of care. RAB participation was completely voluntary and consisted of attendance at monthly one-hour, virtual RAB meetings during the study period, where members were asked to provide suggestions for implementing the CC@BHP intervention, adaptations to the CC@BHP intervention to fit the unit context and workflows, implementation barriers that arose during intervention rollout, and strategies for overcoming these barriers. The overarching goals of the RAB were to enable interactive problem solving during implementation stages to address barriers, streamline the implementation process by identifying areas for intervention adaptation, and to collaborate with academic partners who served as implementation science consultants and monitored implementation progress and outcomes through audit and feedback. The RAB was premised on the concept that successful implementation depends on tailoring implementation strategies to the unique local context, and therefore, the majority of members who participated work in the units where the intervention was implemented. The patients served on these units are predominantly White (65.5%), followed by Black or African American (18.1%), Bi- or Multiracial (8.3%), Latino, or Hispanic (2.5%), and Asian (1.4%). Adolescent patients range in age from 13 to 18 years, and most have private insurance (52%) or Medicaid (44%). Potential members were continuously recruited during the course of the project as we rolled out the intervention across the four units, as well as based on needs identified during the course of the project (e.g., pharmacists were invited as other RAB members discussed their roles in communicating with providers to ensure contraceptives were available). The final RAB roster consisted of 41 members, including 12 physicians, 6 APPs, 4 nurses, 5 program managers of the psychiatric units, 4 quality improvement specialists, 2 pharmacists, 2 parents, 1 adolescent, 1 community health administrator, the Principal Investigator (PI), and 3 implementation science consultants from the partnering academic institution. Of these members, one was identified to also serve as the project champion (an APP), and one member from each of the four units was identified as a unit champion (one physician or APP from each unit). Mean attendance at RAB meetings was 13 individuals (Range 10–15). RAB meetings were audio recorded and transcribed using the transcription service associated with the recording.

We took several steps to ensure equitable participation among RAB members during RAB meetings. First, we used breakout rooms to allow greater space for members to voice their thoughts and opinions within a smaller group during times when potential power imbalances might have been a barrier to open dialog. Members in each small group were intentionally selected to ensure power balances were reduced as much as possible. Second, we encouraged members to anonymously share feedback regarding implementation of the CC@BHP intervention or their thoughts and opinions about the intervention using a web-based sharing platform. Third, at the conclusion of the RAB meetings, we encouraged members to follow-up in a separate email to the study PI or implementation consultants if they did not feel comfortable sharing among the larger group**.**

At the second RAB meeting held in June 2021, we facilitated a semi-structured planning activity with the RAB members involved in the project at that time to gather information on the anticipated barriers to implementation of the CC@BHP intervention. During that meeting, members were asked to report anticipated family-level barriers (i.e., family buy-in, perceptions of the intervention) and practice-level barriers (i.e., time demands, competing priorities) using a collaborative web-based software platform. Each of these barriers was then discussed in more depth during the meeting. Following this meeting, a member of the research team synthesized and organized the barriers according to each of the six core components of the intervention (i.e., sexual history screening, pregnancy intention assessment, assessment of interest in contraception counseling, delivery of the contraception counseling, contraceptive provision, and scheduling follow-up in the adolescent family planning clinic). Next, this same research team member mapped these anticipated barriers to constructs and implementation strategies guided by the Consolidated Framework for Implementation Research (CFIR) and Expert Recommendations for Implementing Change (ERIC) query tool (Damschroder et al., 2009; Powell et al., 2015). Barriers were matched to implementation strategies based on the percentage value of suggested strategies identified in the tool. However, because of the unique and complex context under which this intervention operated, the query tool served as a guide to match barriers to strategies, and hence, strategies did not always represent the highest percentage value. These strategies were used to guide development of an initial implementation blueprint shared with RAB members aimed to steer the early adoption of the CC@BHP intervention on the pilot unit.

#### Stage 2: Development and Implementation

In addition to participation in the monthly meetings, RAB members were also asked to record all activities related to the project that occurred outside of the RAB meetings. These activities were recorded using activity logs and served as our data to identify and specify implementation strategies that we used to guide further development of our blueprint. Activity log data were captured prospectively in RedCap between May 2021 and September 2022. Activity logs are a practical approach to tracking implementation strategies by capturing implementation efforts reported by project members and are one of the most feasible methods for tracking activities related to a project (Bunger et al., 2017; Walsh-Bailey et al., 2021). RAB members were sent weekly reminder emails with a link to the RedCap survey where they were instructed to record the implementation event/activity in as much detail as possible (to specify the implementation strategy), the date and time of the event/activity (the dose), the purpose of the activity (the action), and those involved in the activity (the actors) (Bunger et al., 2017; Proctor et al., 2013). We also requested that members record their name and email address so that the research team could follow up with any questions or to clarify implementation activities as needed. Logs took between 1 and 5 min per log to complete, depending on the level of detail and number of activities reported. To encourage members to include as much detail as possible, de-identified activity log exemplars were visually presented to RAB members during initial RAB meetings. We encouraged members to report any activity they completed that was related to the project so that the academic partners responsible for analyzing the data could clearly map these activities to formal ERIC implementation strategies which were then used to further develop and refine the blueprint.

### Analysis

Monthly activity log data were downloaded from RedCap into an Excel file and reviewed by two research team members (KH and AB). To prepare the data for analysis, we first checked for duplicate activities and combined duplicates into one implementation activity. Duplicates were identified through the description of the activity, activity dates, and the names of individuals who participated in the activity. In addition, if activity descriptions were unclear or lacked sufficient detail, the research team members reached out to the individual who completed the activity log to clarify the activity so it could be matched to a formal implementation strategy. However, activity descriptions were often well detailed by members and, hence, clarifications were rarely needed.

Second, the two coders independently reviewed each implementation activity and purpose and coded them using the Powell et al., (2015) ERIC taxonomy, which is the most recent taxonomy of categories, strategies, and descriptions of strategies available (Powell et al., 2015). Following the coding methods outlined in Bunger et al. (2017), we mapped each activity recorded by the members to implementation strategies from the ERIC taxonomy. Each activity was coded as a discrete implementation strategy. When multiple activities or purposes of activities were recorded by members in one cell block or when activities were related to each other, we broke down each activity based on the context and matched these to a unique implementation strategy; hence, multiple discrete implementation strategies could be identified. For example, one member reported that an in-person discussion was held with her clinician team to provide implementation data regarding the number of consults placed and completed on the unit (two core components of the CC@BHP intervention). Hence, this involved the following strategies: (1) an implementation advisor (KH) who to gathered the implementation outcome data, (2) a designated clinician implementation team meeting aimed to reflect on implementation change efforts and outcomes, and (3) audit and feedback to relay the clinical data back to the team.

The two coders then met to discuss each code and coding discrepancies and finalized the codes using a consensus-agreement approach. Notes were documented throughout the coding process to ensure coding consistency and to reduce variability between the two coders throughout the coding process. Next, the 16 months of coded data were analyzed using univariate statistics in Excel (frequencies, percentages). Frequencies of implementation strategy data were analyzed as a whole, as well as categorically by month and by ERIC category classification.

#### Stage 3: Synthesis and Refinement

The first blueprint that was initially developed based on CC@BHP rollout on IPU was further developed and refined by selecting and operationalizing the most frequently used strategies identified in Stage 2. We organized our blueprint into two main sections using the Lewis et al.’s (2018) blueprint example as a guide: (1) Preparation/Pre-Implementation Phase, and (2) Implementation Phase (Lewis et al., 2018). Each section included overarching goals that needed to be achieved within each phase, implementation strategies to achieve those goals, operationalization of the strategies by specifying action steps needed to carry-out each strategy, the stakeholder responsible for each action step, and provisional deadlines for completing each action step.

**Fig. 1:**
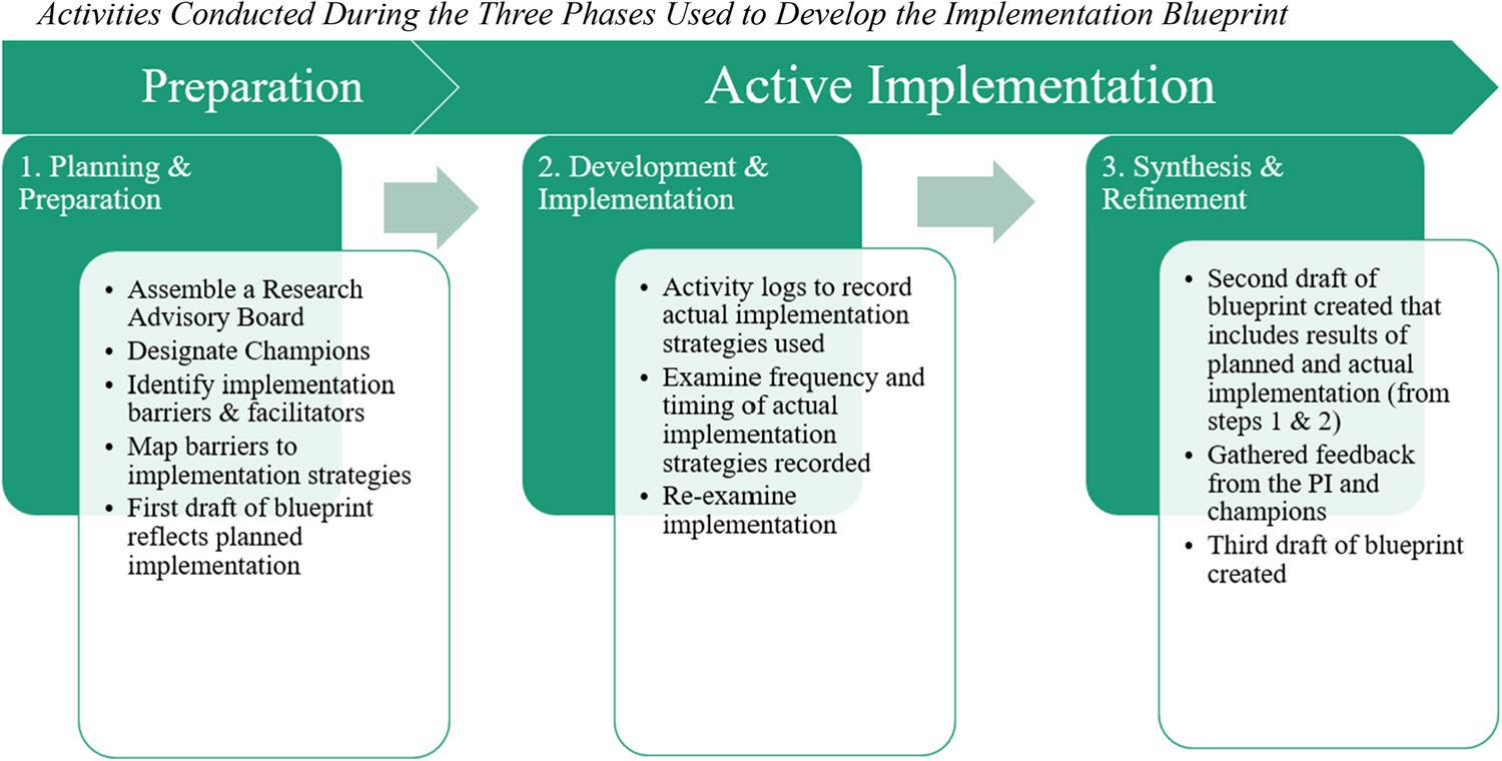
Activities conducted during the three phases used to develop the implementation blueprint

Next, the blueprint was shared with the PI and was refined again based on her feedback, which prioritized strategies based on the unique context of the unit. This blueprint was used as a basis for updating and further developing the blueprint during the course of the project. We consulted with all champions on the final iteration of the blueprint and made changes based on their recommendations. Changes included context-specific strategy prioritization, removal of non-relevant strategies, or inclusion of strategies they deemed necessary for successful implementation of the CC@BHP intervention within this hospital setting. See Fig. 1 for a brief visual depiction of the activities completed during each phase.

### Feedback on the Blueprint

Additional feedback on the usefulness of the blueprint was collected through an informal group discussion with the four champions. Specifically, the first author gathered feedback on what could be done to improve the blueprint (i.e., length, layout, level of detail), *how useful* the blueprint was during intervention implementation, *how often* the blueprint was actually used by champions, and *how* the blueprint was used by the champions to facilitate intervention implementation. Feedback was summarized and is reported below.

## Results

### Stage 1: Planning and Preparation

A total of 19 barriers to implementing the CC@BHP intervention were identified from the facilitated planning activity. Most constructs were situated within CFIR’s ‘Inner-Setting’ domain and included networks & communications, compatibility, relative priority, available resources, and access to knowledge and information. Two constructs were situated in CFIR’s ‘Process’ domain (i.e., planning and patients/consumers) and one construct was situated in CFIR’s ‘Characteristics of Individuals’ domain (i.e., self-efficacy). See Table 1 for a complete list of barriers identified and matched to CFIR constructs. The eight constructs subsequently mapped to eight implementation strategies from the CFIR/ERIC matching tool: (1) Develop a formal implementation blueprint; (2) promote network weaving; (3) identify and prepare champions; (4) develop educational materials; (5) distribute educational materials; (6) promote adaptability; (7) conduct consensus discussions, and (8) patient and family engagement.

**Table 1:** Summary of barriers to CC@BHP implementation identified through the facilitated planning activity

Intervention component	Barriers to component implementation	CFIR constructs
1. Sexual History Screening	• Unclear on the appropriate time to ask patient about sexual history • No standardized workflow to ask about sexual history/conduct sexual history screening • Challenges in provider communications across settings	• Planning • Compatibility • Networks & Communications
2. Assess Pregnancy Intention	• Potential lack of time to complete if intention is 'yes.’ • Location on where to conduct this intervention component is unclear	• Planning • Available resources
3. Assess Interest in Contraceptive Counseling	• Hospital pediatrics providers would not be involved unless there is a patient interested in contraception care. Someone in inpatient should be assessing this and order a consult • Too many consults for providers could make workload high and difficult to assess interest in contraception care. The consults need to be sent over to the right group of providers	• Compatibility • Networks & Communications • Planning
4. Deliver Contraceptive Counseling	• Competing agendas (psychiatric team seeks to stabilize crisis; Hospital pediatrics team seeks to treat physical health) • If demand is greater than the abilities of the advanced practice providers, then the work may be too demanding for the four practitioners • Concerns over quality of contraception care delivery; providers need to have high quality of training and high-quality delivery. Currently, providers do not feel prepared to deliver contraception care, including how to discuss with parents and provide parents with accurate information • Need to get the ‘green light’ from psychiatry that the patient is fully oriented to engage in contraception care; where will this happen? Is there a designated space for this that will keep patients’ privacy and confidentiality? • During day, patients have a lot of psychiatry programming that will need to be worked around	• Compatibility • Relative priority • Available resources • Self-efficacy • Planning
5. Provide Contraceptive	• Lack of knowledge of confidentiality laws and clinical pathways to maintain confidentiality with documenting, ordering/discussing laboratory results, medications, referrals • Contraceptives are currently not readily available through the pharmacy, which delays contraceptive provisions, particularly with short hospital stays	• Access to knowledge and information • Panning • Relative priority
	• Patient may be engaging in therapies when hospital pediatrics provider is available to provide counseling/contraceptive	
6. Schedule Follow-up in BC4Teens Clinic when indicated	• Patients on YSCU usually have a short and more predictable length of stay, but other units may have patients who have infinite length of stays, which may complicate follow-up scheduling • Concerns over patients not showing up to follow-up visits	• Patients/customers

Each of these implementation strategies (except for developing a formal implementation blueprint, which was the objective of this study), was operationalized in the pilot blueprint with specific action steps, personnel responsible for completing the action steps and their role, and a timeline for completion. Each of these components in our pilot blueprint was included to ensure the clinical team on each unit and stakeholders were prepared for intervention rollout. For example, one of the overarching goals we identified as necessary to our project’s Preparation/Pre-Implementation Phase was to train leadership to support implementation. The strategy to accomplish this goal was ‘Identify and Prepare Champions,’ which was operationalized in the specific action steps as follows: (1) identify the champion(s) during team planning meetings and (2) train the unit champions using the champion training document developed for this project (Supplemental File 2). Another implementation strategy identified at this stage was ‘involve patients/customers.’ We operationalized this strategy in the action steps as follows: (1) Obtain RAB member input for how to involve patients and family members and (2) Solicit feedback from RAB members for ensuring patient follow-up visits are scheduled and attended. These action steps resulted in the inclusion of one patient and one family member as RAB members as well as solutions to ensuring that follow-up visits were scheduled and attended (i.e., complete on-site referrals for follow-up prior to patient discharge, and provide text reminders to patients prior to follow-up appointments), which were subsequently added into the blueprint. In addition, throughout the course of our RAB meetings prior to our intervention launch date, we recognized that the ‘promote adaptability’ strategy needed to be more concrete due to the complexity of our multicomponent intervention as well as the complexity of operations on our units. Therefore, we operationalized ‘promoting adaptability’ by creating a process flow map for the pilot unit to aide in guiding, streamlining, and promoting adaptations to the intervention to fit the implementation process within the unique workflow of the unit. Each of the other goals, strategies, action steps, individuals, and timelines for our blueprint are in Supplemental File 3.

### Stage 2: Development and Implementation

A total of 192 activity log entries were recorded by 16 different RAB members over the 18-month data collection period, which mapped to 37 unique implementation strategies. The majority of implementation activities were situated in the ‘Train and Educate Stakeholders’ ERIC category (28.3%), followed by 20.1% in ‘Use Evaluative and Iterative Strategies,’ 16.1% in the ‘Develop Stakeholder Interrelationships’ category, 15.7% in the ‘Adapt and Tailor to Context’ category, 11% in ‘Change Infrastructure,’ 3.5% in ‘Engage Consumers,’ 2.4% in ‘Support Clinicians,’ 1.6% in ‘Provide Interactive Assistance,’ and 1.2% in ‘Utilize Financial Strategies.’

**Fig. 2:**
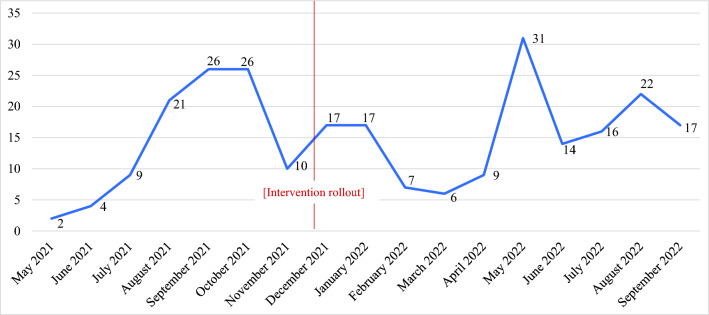
Total strategies used each month

The total number of implementation activity log entries increased over the course of the first 6-month planning period, followed by a stark drop of activities recorded immediately prior to intervention rollout on our pilot unit on December 1, 2021. However, implementation activities also had peaks in May and July 2022, during later phases of implementation. See Fig. 2.

**Fig. 3:**
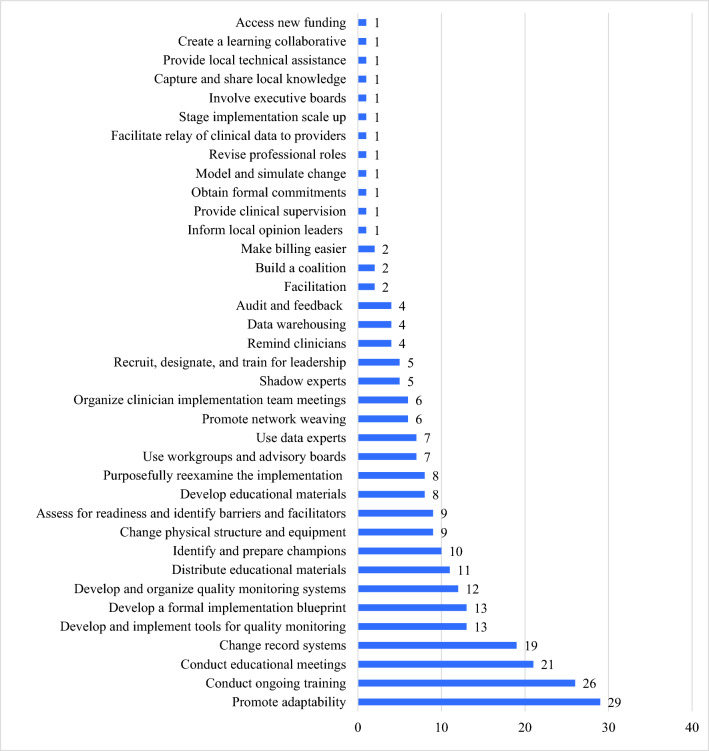
Frequency and type of implementation strategies used over the 18-month period

Of the overall activities recorded by members during the project period, a total of 29 mapped to ‘promote adaptability,’ which was the most frequently identified implementation strategy. Other frequently identified strategies included ‘conduct ongoing training’ (*n* = 26), ‘conduct educational meetings’ (*n* = 21), and ‘change record systems’ (*n* = 19). See Fig. 3 for the complete list of implementation strategies identified.

In Quarter 1 of the project leading up to intervention rollout, most activities focused on training and educating stakeholders, as well as developing stakeholder interrelationships. Namely, providers were being trained on how to deliver the intervention, including how to approach patients about contraception counseling and what language to use during counseling sessions, how to provide certain types of contraceptives, and completing etonogestrel placement training. Developing stakeholder relationships aimed to prepare stakeholders for implementation of the CC@BHP intervention through network weaving, where champions held meetings and small group discussions with providers to promote the intervention and the importance of adopting the intervention to improve the quality of clinical care.

In Quarter 2, most implementation activities shifted to the use of evaluative and iterative strategies, as well as changing infrastructure. Specifically, critical to the periods leading to intervention rollout was the development and organization of quality monitoring systems, which for our project, included changing electronic health record infrastructure to accommodate providers’ preferences for recording contraception orders, completions, and contraceptive provisions. In addition, because of the lack of designated space and contraceptives needed to deliver the intervention, providers frequently recorded activities related to clearing out and setting up rooms designated to CC@BHP delivery, which included hanging educational posters about contraception on the walls in the room(s), preparing pamphlets to send home with patients and/or caregivers, and stocking contraceptives on the units.

In Quarter 3, activities shifted again to a focus on adapting and tailoring to the context, as well as continuing to utilize evaluative and iterative strategies. Specifically, as we rolled out intervention on each unit, strategies focused on refining the flow maps and blueprint to work with the unit workflows. For example, this involved adjusting electronic health records to include a section for confidential notes or adjusting contraception consultation and/or procedure billing charges. In addition, implementation strategies included providing audit and feedback, facilitation, organizing clinician team meetings, purposively reexamining implementation of the CC@BHP intervention, and facilitating relay of clinical data to providers.

**Fig. 4:**
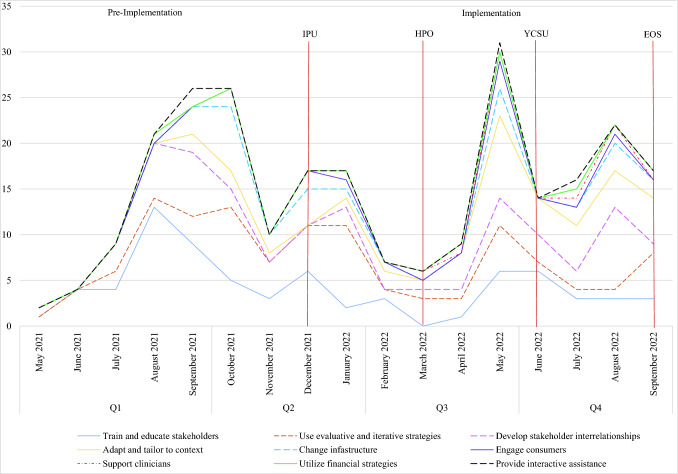
Frequency of ERIC categories per quarter. *IPU* inpatient psychiatric unit, *HPO* hospital pediatrics observation unit, *YCSU* youth crisis stabilization unit, *EOS* psychiatric crisis department extended observation care unit. *Note* Implementation begins at IPU

Quarter 4 included strategies focused on continuing to develop stakeholder interrelationships as the intervention was rolled out across the other units, adapting and tailoring the intervention to these unit contexts, and training providers who were new to the hospital or project on how to deliver the intervention. See Fig. 4 for the breakdown of how ERIC categories were represented over the 18-month project period.

### Stage 3: Synthesis and Refinement

The final blueprint consisted of 16 goals linked to 10 strategies for the Pre-Implementation Phase and 6 strategies for the Implementation Phase. Goals in each phase were modeled after the main ERIC categories, and specific ERIC implementation strategies to accomplish each goal.

### Blueprint Pre-implementation Phase

During Pre-Implementation, the goals and strategies focused on preparing champions and clinical teams for intervention rollout. Our first goal was to train leadership to support implementation, which we did by identifying and preparing champions. Specifically, the PI and implementation consulting team identified champions during project planning meetings and trained them using a champion training document and individual meetings with the PI. Our second goal was to promote stakeholder interrelationships to enhance communication between units and clinical teams implementing the intervention, and to promote positive team culture, climate, and attitudes toward the intervention. We used two specific implementation strategies to build relationships: network weaving and organizing clinician implementation team meetings. The specific action steps included champion-led small group meetings and individual discussions with clinical staff to spread the innovation, and engaging members from multiple units in RAB meetings to promote cross-unit collaboration. Another goal was to assess readiness for implementation on each unit to stage implementation scale-up. Action steps included meetings among champions and PI meet to discuss intervention rollout on the next unit, assessing and addressing potential implementation barriers, and ensuring that the unit had necessary resources (i.e., contraceptives are stocked on the units, a room is available for intervention delivery). The Pre-Implementation Phase of the blueprint was also consolidated into a brief checklist for simplicity, which served as a final confirmation of unit preparedness immediately prior to intervention rollout (Supplemental File 4).

### Blueprint Implementation Phase

During Implementation, our main goal was to ensure successful implementation of the CC@BHP intervention as planned. One of these strategies was to purposively re-examine implementation. To do so, unit champions addressed clinical team members’ concerns and questions, assessed implementation barriers that arose throughout implementation, and adjusted workflows accordingly. Another strategy for ensuring successful implementation was to facilitate relay of clinical data back to the implementation teams. Specifically, quality improvement personnel and a member of the implementation research team pulled implementation outcome data, developed data visualizations, and relayed the information back to the clinical implementation teams. Another major goal during implementation was to ensure that the intervention fit the unique contexts of each unit, which meant that promoting adaptability was a necessary strategy. To promote adaptability, the unit champions and clinician teams worked together to identify where adaptations needed to occur, track adaptations in activity logs, and revise or refine the flow map as necessary. To accomplish our goal of monitoring the fidelity of the CC@BHP intervention, we sent reminders and supported clinicians. Specifically, an advance practice nurse with expertise in contraception care observed individual implementers each month and tracked fidelity to the intervention components according to our pre-specified fidelity metrics. The trainer used a published checklist to measure quality of contraception care and reviewed the observed findings with the implementing clinicians (Reproductive Health National Training Center, 2022). The implementers reported on their calendars a specific date each month for fidelity monitoring by this trainer to ensure accountability and to contact the trainer to arrange the observed counseling sessions. See Supplemental File 3 for our final implementation blueprint consisting of each of the goals, strategies, action step, person(s) and role(s), and timelines for completion. A brief visual depiction of the activities from each of these three stages is provided in Fig. 1.

### Feedback on the Implementation Blueprint

Our final implementation blueprint demonstrated strong face validity according to the project champions, who found the blueprint useful for implementation of the CC@BHP intervention. Specifically, one of our champions discussed the importance of this blueprint to plan for intervention rollout on each unit by thinking through each of the steps necessary for staging the rollout process. Notably, champions also discussed that the blueprint can be used as a training and orientation guide to the intervention for clinical staff, as well as new clinicians who may be hired during intervention implementation. Specifically, they considered the blueprint a necessary first step in the implementation process to aid in efficiency and training.

Most notable was the role of the primary project champion in translating the blueprint for other clinicians to use since many clinical team members in our study did not have the time to review each step in the document. Our primary project champion who was most familiar with the blueprint reported translating each goal and strategy into smaller, actionable, and more digestible steps for clinicians and other unit champions. In addition, champions reported that most clinicians are not familiar with the nomenclature used in implementation science, and therefore, the champion training document in conjunction with guidance from the academic partners was helpful to becoming familiar with key implementation science terms (i.e., what is an implementation strategy), how to use implementation strategies to drive the implementation process, and how to adapt these strategies to meet the needs of the clinicians. In addition, participants reported that personal characteristics of the champions matter. Specifically, they reported that champions must have time to be dedicated to the project, understand, and believe in the usefulness of the intervention, and have strong leadership skills, strong communication skills, and the ability to work well on interdisciplinary teams. Some champions reported that future blueprints could potentially be improved by reformatting the layout from table format into diagrams to aide in visualizing the action steps and processes needed to guide intervention rollout.

## Discussion

The objective of this study was to develop an implementation blueprint to stage scale-up of the CC@BHP intervention across hospital units. This resulted in the development and refinement of an implementation blueprint tailored to our specific context, with input from unit champions. In a study conducted by Lewis et al. (2018), the authors described a five-step process for developing an implementation blueprint in one youth residential setting (Lewis et al., 2018). Our work builds on this example by describing the development of an implementation blueprint, refining it based on observed implementation strategies, and pilot testing it across multiple fast-paced inpatient units in the hospital. In addition, our work adds to the implementation science literature by describing how we used a collaborative approach to develop and refine the blueprint with healthcare providers. In turn, this allowed us to create a more specific blueprint tailored to situational factors, contexts, and the specific needs of providers, their workflows, and the patients they treat. Utilizing a more collaborative approach to implementation strategy development can potentially help increase stakeholder engagement and responsiveness to the intervention and processes for implementation. These methods could be replicated across similar complex environments to improve the planning, reporting, and replication of implementation efforts. Future research is needed to investigate how this implementation blueprint affected implementation outcomes, such as increasing adoption and reach of the clinical intervention, and contributing to fidelity to the intervention.

### A Systematic Process for Developing and Refining a Blueprint

Our work builds on prior reports of blueprint development by presenting a systematic process for developing and refining an implementation blueprint using existing tools and resources from the larger implementation science field. Specifically, we used the CFIR-ERIC matching tool to identify strategies that addressed barriers to implementation that were identified by our Research Advisory Board. Other studies have demonstrated the usefulness of the CFIR-ERIC matching tool (Shin et al., 2022; Weir et al., 2021), although the extent to which this tool generates the most effective and comprehensive set of implementation strategies warrants additional testing. In our work, we complemented our use of the CFIR-ERIC matching tool with an activity log to capture strategies used in real time. Together, these methods captured a range of implementation strategies used over time which reflects the intensive nature of implementation. Consistent with other observational studies of implementation strategies, our work illustrates the emphasis on building relationships and communication as necessary for successful implementation of the intervention (e.g., Boyd et al., 2018; Bunger et al, 2017). More research is needed to understand the key ingredients of communication and relationship-building strategies on intervention adoption in health settings (Bartley et al., 2022).

While anticipatory barrier and strategy mapping are an important first step for developing blueprints, we found that it was insufficient; prospective strategy tracking was necessary for refining the blueprint in a way that would allow other units and sites to replicate it. Our strategy-tracking efforts with activity logs identified a greater number and diversity of implementation strategies than our planning work with the CFIR-ERIC matching tool. This highlights the practical utility of strategy-tracking methods (Walsh-Bailey et al., 2021), especially to capture the unplanned implementation strategies often used to respond to emerging issues (Smith et al., 2022). To develop blueprints that would allow replication and scale up in multiple sites, both anticipatory barrier-strategy identification and real-time strategy-tracking methods can be used in complementary ways.

### Using Blueprints to Replicate Implementation

Replicating implementation across inpatient units generated several unique insights. First, by tracking strategies used during replication on other units, we produced substantial operational detail about strategies for promoting the adaptability of the intervention and tailoring strategies to fit the context. The four hospital units in this study each had unique workflows, different individuals responsible for implementing the intervention, and different patient needs, which subsequently required that implementation strategies were operationalized according to each of these contexts. For example, ‘change record systems and develop tools for quality monitoring,’ which was one strategy used in the Preparation/Pre-Implementation phase, was operationalized differently across units based on provider preferences. Specifically, providers on each unit designed a unique smart phrase (i.e., a section of pre-generated text by typing a dot (.) and then selecting the matching phrase to place into the clinical note) to fit their preferences for capturing the adoption of different components of clinical intervention. As another example, ‘promote adaptability’ was used in the Implementation Phase and was operationalized as providers’ adaptations to the intervention flow maps to fit the context of the unit. Hence, flow maps which guided the implementation of the CC@BHP intervention were tailored to each unit. This study highlights the inherent differences in the operationalization of implementation strategies necessary to fit unique contexts and underscores the need for future studies to move beyond reporting the standardized definitions of implementation strategies to also detailing their operationalization. In turn, this will allow further development and specification of implementation strategies to help delineate mechanisms through which these strategies operate.

Our work also offers insights about how blueprints are used during implementation. While many professionals and stakeholders provided input during planning, and by offering detail about strategies used in the activity logs, the actual blueprint was primarily used by champions during replication. Our results illustrate how champions used the blueprint to plan for and communicate the plan to implementation teams. The unit champion was a key player in our work and translated the blueprint into practice (although we acknowledge that facilitators, technical assistance providers, or other types of implementation support practitioner might play this role in other settings). In our work, we developed a champion training document describing how to use the blueprint which our champions found to be helpful. This highlights the importance of educating and supporting champions (or other implementation support practitioners) to use blueprints.

As the implementation practice and research community refines blueprint development processes and products, it will also be important to consider champion (or other blueprint user) preferences for formatting, content, and other features that influence interpretability. Depending on disciplinary training, experience, and learning/style, some champions might prefer visual blueprints with diagrams to illustrate flow of each step. For complex interventions like ours that require multiple strategies, it might also be important to bundle strategies (by domain, goal, or user) to enhance the digestibility of the blueprint.

### Limitations

This study is not without limitations. First, although we utilized multiple methods to capture implementation activities as well as implementers’ feedback on and use of the blueprints (i.e., activity logs, RAB, operations meetings, Champion meeting), it is possible that our methods did not fully capture all implementation activities completed by providers over the 16-month data collection period. Specifically, of the 41 RAB members, only 16 reported on any implementation activities, likely missing other strategies that were completed but not reported. In addition, providers were asked to self-report activities each week, which may have resulted in recall bias., For example, it is possible that the stark drop-in recorded implementation activities immediately prior to the implementation start date may have been affected by seasonal events occurring around the time of year we began implementation rollout (i.e., December 1st, 2021). However, it is also possible that the planning stage of the project required more activities to be conducted, and hence, more strategies were recorded during the project planning stage. Another potential limitation was the variation in RAB attendance each month, which could have impacted identification of barriers and implementation strategies during the pre-planning stages, or discussions on what strategies were most useful during implementation. In turn, this could have affected which strategies took priority over others during refinement of the blueprint. In addition, we developed the CC@BHP blueprints within the context of a large quaternary children’s hospital that had a dedicated hospitalist service to care for the medical needs of adolescent patients admitted for psychiatric problems. While the process of developing the blueprint is generalizable to other settings, this blueprint will need to be tailored to the unique structures, cultures, and resources of different healthcare settings. Future research should investigate whether the use of blueprints adapted for other settings can enhance efficiency and/or speed of implementation (Proctor et al., 2011). Similarly, another limitation is that the ERIC taxonomy does not have any strategies that explicitly focus on health equity, and hence, the strategies specified in our blueprint do not have an explicit health equity focus. Future research is needed to better understand how these strategies can be adapted and leveraged to increase health equity among marginalized and vulnerable populations.

## Conclusions

A multidisciplinary team successfully implemented a contraceptive care access intervention in four inpatient psychiatric units in a children’s hospital using a blueprint to guide implementation. Our methods for blueprint development, utilization, and refinement can be applied in other settings to either tailor our blueprint to assist in scaling out the intervention to other hospital settings or can be used to develop blueprints for increasing the uptake and scale other interventions in hospital settings. Specifically, the blueprint can serve as a template for other teams who want to replicate this work in their hospital. Future research should test whether implementation blueprints enhance intervention adoption, reach, and effectiveness.

## Supplementary Information

Below is the link to the electronic supplementary material.

Supplementary file1 (DOCX 40 kb)

Supplementary file2 (DOCX 29 kb)

Supplementary file3 (DOCX 39 kb)

Supplementary file4 (DOCX 22 kb)

## Data Availability

These data contain confidential information about employee practices, and patients, therefore, they are not publicly available.

## Abbreviations

APPs: Advance practice providers
CDC: Centers for Disease Control and Prevention
CFIR: Consolidated Framework for Implementation Research
CC@BHP: Contraception Care at Behavioral Health Pavilion
HER: Electronic Health Records
ERIC: Expert Recommendations for Implementing Change
PI: Principal Investigator
RAB: Research Advisory Board
STROBE: Strengthening the Reporting of Observational Studies in Epidemiology
