# Supplementary material for: Development of an Implementation Blueprint to Scale-Up Contraception Care for Adolescents with Psychiatric Conditions in a Pediatric Hospital
Source: Glob Implement Res Appl. Author manuscript; Available in PMC 2024 Jan 30. (PMC10827339; doi:10.1007/s43477-023-00082-7)
Supplement: Supplemental File 2 [file NIHMS1952276-supplement-Supplemental_File_2.docx]

**Supplemental File 2**

*Champion Training Document*

**Definition of the ‘champion’**

Champions for intervention implementation are “individual(s) who dedicate themselves to supporting, marketing, and driving through an implementation, overcoming indifference or resistance that the intervention may provoke in an organization” (Powell et al., 2015, page 9).

The goal of incorporating a champion is to help promote change in the way things are done to the way things should be through *leadership*.

**Roles of the CC@BHP Project Implementation Champion**

For our CC@BHP intervention, the following roles of the project champion include but are not limited* to:

1. **Building rapport with implementing staff:**
   - Identify as the CC@BHP project champion.
     - Center oneself as the point-of-contact for the CC@BHP intervention within and throughout NCH.
   - Have a physical presence among the implementing practitioners.
   - Provide encouragement and motivation to peers.
2. **Promoting the CC@BHP intervention within NCH through**:
   - Informal discussions about CC@BHP with practitioners and staff.
   - Modelling positive attitudes, enthusiasm, and personal belief in the CC@BHP intervention.
     - Importance and worthwhileness of CC@BHP.
     - Benefits to patients.
3. **Providing peer-to-peer coaching on CC@BHP intervention implementation.**
   - Walk through CC@BHP activities with providers as needed.
   - Help to answer questions on intervention delivery.
4. **Delivering informal education to peers as needed.**
   - Reminders about the intervention and its components.
   - Provide information about contraception and/or the intervention to peers (questions can also be relayed from the project champion to the Principal Investigator).
5. **Overseeing general implementation activities of CC@BHP.**
   - Ensure the intervention is being implemented.
     - May require informal or formal check-ins with implementing staff.
   - Help to optimize the workflow to fit with the intervention components.
     - Promote adaptation.
   - Monitor if goals are being met.
   - Ensure availability of supplies and materials.
   - Lead development and implementation of electronic health record fields, workflows, and related education.

*Additional activities not listed here will emerge throughout the implementation process and should be logged using the online Activity Log. This will help us to define the role more clearly in publications and for other hospitals who wish to implement the CC@BHP intervention.

The following literature provides additional information on the role of project champions as an implementation strategy.

1. Bonawitz, K., Wetmore, M., Heisler, M., Dalton, V. K., Damschroder, L. J., Forman, J., Allan, K. R., & Moniz, M. H. (2020). Champions in context: Which attributes matter for change efforts in healthcare? *Implementation Science*, *15*(1), 62. <https://doi.org/10.1186/s13012-020-01024-9>
2. Goedken, C. C., Livorsi, D. J., Sauder, M., Vander Weg, M. W., Chasco, E. E., Chang, N.-C., Perencevich, E., & Reisinger, H. S. (2019). “The role as a champion is to not only monitor but to speak out and to educate”: The contradictory roles of hand hygiene champions. *Implementation Science*, *14*(1), 110. <https://doi.org/10.1186/s13012-019-0943-x>
3. Miech, E. J., Rattray, N. A., Flanagan, M. E., Damschroder, L., Schmid, A. A., & Damush, T. M. (2018). Inside help: An integrative review of champions in healthcare-related implementation. *SAGE Open Medicine*, *6*, 2050312118773261. <https://doi.org/10.1177/2050312118773261>
4. Powell, B. J., Waltz, T. J., Chinman, M. J., Damschroder, L. J., Smith, J. L., Matthieu, M. M., Proctor, E. K., & Kirchner, J. E. (2015). A refined compilation of implementation strategies: Results from the Expert Recommendations for Implementing Change (ERIC) project. *Implementation Science: IS*, *10*, 21. <https://doi.org/10.1186/s13012-015-0209-1>
5. Shea, C. M. (2021). A conceptual model to guide research on the activities and effects of innovation champions. *Implementation Research and Practice*, 2, 2633489521990443. <https://doi.org/10.1177/2633489521990443>
