# Supplementary material for: Development of an Implementation Blueprint to Scale-Up Contraception Care for Adolescents with Psychiatric Conditions in a Pediatric Hospital
Source: Glob Implement Res Appl. Author manuscript; Available in PMC 2024 Jan 30. (PMC10827339; doi:10.1007/s43477-023-00082-7)
Supplement: Supplemental File 3 [file NIHMS1952276-supplement-Supplemental_File_3.docx]

**Supplemental File 3**

*Implementation Blueprint*

**Implementation Blueprint**

This is the master implementation blueprint for the CC@BHP intervention at Nationwide Children’s Hospital. This document contains instructions for how to roll-out CC@BHP and was developed based the coded activity logs and implementation strategies, as well as consensus discussions with unit champions and the Principal Investigator. The blueprint was updated and refined over the project period. *Adaptations can and should be made* *to this blueprint as needed* *based on the unique clinical context.*

| **I. Preparation/Pre-implementation Phase** | | | | |
| --- | --- | --- | --- | --- |
| **Goal** | **Strategy** | **Action Steps** | **Person [Role]** | **Timeframe** |
| 1. Evaluate preparedness and readiness for implementation | Assess for readiness by identifying barriers and capitalize on facilitators to implementation | - Communicate by email to identify barriers and facilitators to CC@BHP implementation and help ensure preparedness and readiness for implementation. - Hold a unit meeting to identify any potential barriers to implementing the CC@BHP intervention and assess for readiness. | Unit champions and providers responsible for delivering the intervention.  [Unit champions will communicate regularly with providers and with other unit champions to trouble shoot problems, ensure readiness to implement the intervention, and prepare the unit for implementation.] | Month 1 |
| 2. Clinician and provider coordination of care | Consensus discussions | - Determine the best times to approach the patient about the CC@BHP intervention when they arrive on unit. (Ensure enough time to order contraception care and provisions prior to patient discharge.) | Champions | Month 1 |
| 3. Train leadership to support implementation | Identify and prepare champions | - Identify project champions during implementation team planning meetings; discuss with other members of CC@BHP team. - Identify unit champions. - Train the unit champions using the champion training document and meeting with Principal Investigator (PI).   *A psychiatry and pediatrics physician champion plus an advance practice provider project champion should be identified and trained. A project champion may also be a unit champion. | Principal Investigator, academic consultant, pediatric project physician champion, psychiatry project champion, advance practice provider project champion  [Project champions are responsible for assisting with the overall project, and successful intervention rollout.] | Months 1 – 2 |
| 4. Ensure implementation outcome data can be tracked. | Change record systems and develop tools for quality monitoring | - Determine electronic health record (EHR) and develop smartphrases tailored to providers’ preferences. - Submit EHR change requests ASAP. - Provider-to-provider communications to enforce use of trackable measures (Smartphrases, SmartLists, Consult Orders, Contraception Ordersets, and Hospital Pediatrics Consultation Service). | Champions and quality improvement specialist | ASAP |
| 5. Promote stakeholder interrelationships to enhance communication, positive team culture, climate, and attitudes. | Network weaving | - Hold individual and small group discussions with clinical staff to promote the CC@BHP intervention and the importance of the intervention to providing high quality clinical care. | Unit Champion(s)  [Unit champions will promote relationship building and enhance team knowledge through meetings and individual discussions.] | Ongoing |
| 6. Train providers to enhance their knowledge and self-efficacy to deliver each intervention component. | Distribute educational materials and conduct training | - All providers will engage in training on the entire CC@BHP intervention. | CC@BHP trainer | Months 1 – 2  Ongoing for new hires and fellows. |
| 7. Enhance knowledge of CC@BHP among clinicians on unit team | Conduct educational meeting | - Hold a unit meeting to discuss CC@BHP intervention and goals. - Present flow map to team. - Introduce electronic health record (EHR) Smartphrases and trackable measures to clinicians, clarify workflow, and answer questions prior to rollout. - Present activity logs to team so they know to track implementation efforts. | PI, champions, and all members of the unit implementation team should attend. | Months 1 – 3 |
| 8. Infrastructure changes to ensure unit is ready for implementation | Change physical structure and equipment | - Engage pharmacy to order contraception and to ensure contraceptives are available on the unit. - Ensure etonogestrel kits are on the unit. - Post etonogestrel set-up diagrams in treatment rooms. - Advanced practice provider will maintain inventory of etonogestrel implant procedure supplies. | Champions, unit coordinator | Month 4 |
| 9. Ensure CC@BHP intervention process is adapted to the unit workflow | Implementation blueprint refinement and promote adaptability | - Create or adapt the flow map for the unit. | PI, academic consultant, and champions.  [Adapt flow map according to unit workflows and practices.] | Two weeks prior to intervention rollout on each unit. |
| 10. Readiness for Implementation | Stage implementation scale up | - Champions to meet with PI a final time to discuss plans for rolling out CC@BHP on unit. - Final review of flow map to ensure preparedness for implementation. - Ensure that barriers have been addressed. | Champions and PI | One week prior to intervention rollout |
| **II. Implementation Phase** | | | | |
| **Goal** | **Strategy** | **Action Steps** | **Person and Role** | **Timeframe** |
| 1. Promote stakeholder interrelationships to ensure successful implementation | Organize clinician implementation team meetings | - Clinical team operations meetings to discuss implementation challenges. | Champions and providers  [Champions will hold regular clinical team meetings with the unit to ensure implementation success and manage any issues that arise during implementation. | Monthly |
| 2. Monitor fidelity | Remind and support clinicians | - Observe subset of CC@BHP consults using the Contraceptive Counseling and Education Checklist (Reproductive Health National Training Center, 2022) and track fidelity. - Coordinate at least one observation per clinician per month. - Reminder e-mails sent out mid-month to secure observations. - CC@BHP trainer provides feedback using the Contraceptive Counseling and Education Checklist. | Advanced practice provider, CC@BHP trainer, intervention providers | Monthly |
| 3. Ensure CC@BHP continues to fit the clinical context | Promote adaptability | - Unit champions + clinic staff on the unit can identify where adaptations needed to occur during implementation to fit the clinical context. - Track adaptations in activity logs. - Involve informatics as needed. - Revisit and refine flow map. | Champions and providers | Ongoing |
| 4. Ensure successful implementation | Purposively reexamine implementation | - Address questions and concerns as they arise from the team. - Address any barriers to implementation | Champions | Ongoing |
| 5. Ensure successful implementation and that implementation outcomes are being met. | Facilitate relay of clinical data | - Involve informatics and quality improvement team to pull data from the electronic health record. - Use EHR data to create charts on the implementation outcomes. - Unit champions will send out a monthly email to relay data back to providers about implementation outcomes. | Champions, quality improvement specialist, academic partner | Monthly |
| 6. Ensure implementation outcomes are being met and promote sustained behavior change | Audit and feedback | - Discuss implementation outcome data for each unit with clinical teams and Research Advisory Board (RAB)*, including what is going well and what needs to be changed. | PI, Champions, RAB members | Monthly |

*Note: Members volunteered to participate on the RAB. The volunteers included clinical unit physician leaders and healthcare providers (physicians, advanced practice providers (APPS), nurses) of these units where we implemented the CC@BHP intervention, as well as implementation scientists of the partnering academic institution. In addition to these volunteers, we also reached out to additional individuals to ask for their participation on the RAB based on needs identified over the course of intervention rollout. The final RAB comprised 41 members, including 12 physicians, 6 APPs, 4 nurses, 5 program managers of the psychiatric units, 4 quality improvement specialists, 2 pharmacists, 2 parents, 1 adolescent, 1 community health administrator, the Principal Investigator, and 3 implementation science consultants from the partnering academic institution. Importantly, the project and unit champions were all RAB members. Therefore, when considering who should comprise the RAB, it was important consider context-specific needs as well as availability of members with specialized skills in clinical care delivery, expertise in data management (for quality improvement reports and implementation outcomes), and implementation science.
