# Supplementary material for: Development of an Implementation Blueprint to Scale-Up Contraception Care for Adolescents with Psychiatric Conditions in a Pediatric Hospital
Source: Glob Implement Res Appl. Author manuscript; Available in PMC 2024 Jan 30. (PMC10827339; doi:10.1007/s43477-023-00082-7)
Supplement: Supplemental File 4 [file NIHMS1952276-supplement-Supplemental_File_4.docx]

**Supplemental File 4**

*Final Implementation Checklist*

**Pre-Implementation Plan Final Checklist**

[Unit Name]

To Do: (Check off when complete)

- Submit EPIC change request for smart phrase for documenting routine consult offer
- EPIC change request for smart phrase for documenting routine consult offer is in production
- Finalize implementation outcome plan with Quality Improvement lead
- Determine that [unit] Contraception Care consult order is compliant with billing
- Make sure Nexplanon kits are on any unit where CC intervention might occur
- Hang Nexplanon set up photo in clinical rooms where CC intervention might occur
- Ensure team coordinator has ordered supplies/prepped for future supply needs
- Make sure nurses and APPs have the contraceptive keychain
- Ensure all providers know to routinely offer contraception consultation to eligible patients
- Ensure all providers know to use the smart phrase for documenting each consult offer and what the phrase means (send email reminder)
- Unit champions to ensure recurring meetings with project champions are on calendars
- Schedule recurrent team meetings with clinical staff who are implementing the intervention
- Finalize the flow map for the team, including how communication between providers will occur
- Distribute flow map to the team and unit staff members
- Ensure the team has the Excel consult tracker from project champion and knows to immediately input consult tracking data and how to do so
- Ensure the team is aware of Activity Logs and how to fill in all activities related to the CC@BHP intervention
- Ensure the team is on the email list for Activity Logs
- Send final email to the clinical team and staff that implementation launch date is December 1, 2021
